# LINE-1 Methylation Status in Multiple Sclerosis Patients Is Associated with Changes in Folate Metabolism

**DOI:** 10.32607/actanaturae.27579

**Published:** 2025

**Authors:** E. A. Tsymbalova, E. A. Chernyavskaya, G. N. Bisaga, A. Y. Polushin, E. I. Lopatina, I. N. Abdurasulova, V. I. Lioudyno

**Affiliations:** FSBSI “Institute of Experimental medicine”, Saint Petersburg, 197022 Russia; Almazov National Medical Research Center, Saint Petersburg, 197341 Russia; FSBEI HE “Academician I.P. Pavlov First St. Petersburg State Medical University” of the Ministry of Healthcare of Russian Federation”, Saint Petersburg, 197022 Russia

**Keywords:** methylation, LINE-1, multiple sclerosis, homocysteine, folate metabolism, C677T polymorphism

## Abstract

The disruption of epigenetic regulation and the development of abnormal DNA methylation patterns are crucial steps in the pathogenesis of neurodegenerative diseases. Methylation alterations in multiple sclerosis (MS) patients may contribute to the dysregulation of gene expression linked to the regulation of inflammation, myelin production, and the preservation of the integrity of the myelin sheath. The possibility that epigenetic alterations could be reversed provides a rationale for studying their mechanisms. In this study, we evaluated the methylation status of LINE-1 retrotransposons in the peripheral blood cells of patients with MS and healthy controls. In healthy individuals, LINE-1 methylation levels were observed to decrease with advancing age. MS patients exhibited a positive correlation between LINE-1 methylation and MS duration. The study indicates that the level of LINE-1 methylation is notably higher in progressive MS compared to the remitting type. LINE-1 methylation variations in MS patients were observed to be associated with the serum levels of homocysteine and vitamin B9, and dependent on the genotype for the C677T polymorphism of the *MTHFR *gene as well. The data obtained point to the contribution of the C677T polymorphism to the appearance of epigenetic disorders in MS development and suggest that hypermethylation may be mediated by disruptions in the folate metabolism that accompany MS.

## INTRODUCTION

Multiple sclerosis (MS) is a chronic autoimmune demyelinating disease that is associated with progressive neurological symptoms and patient disability. The etiology of MS is based on both genetic susceptibility and external factors that initiate the pathological process [1, 2]. Progress in the study of genome modifications has revealed a more intricate picture of MS pathogenesis, encompassing epigenetic factors such as alterations in DNA methylation. Alterations in the susceptibility to external factors and the increased disease development risk may be attributed to irregularities in gene expression that stem from hypo- or hypermethylation of regulatory regions within the genome [3].

A whole-genome analysis indicates notable variations in DNA methylation profiles among individuals with MS compared to the control group [4, 5, 6]. In the progressive course of MS, differentially methylated sites are predominantly hypermethylated [7]. The detectable alterations in DNA methylation influence the mechanisms governing blood-brain barrier (BBB) permeability, the regulation of the immune-inflammatory response, the processes of mature myelinating oligodendrocyte formation, and the maintenance of myelin sheath stability [6, 8, 9]. A relationship has been identified between LINE-1 hypermethylation and a greater likelihood of clinical disease activity through the analysis of global genome methylation, assessed by examining LINE-1 retrotransposon methylation in blood cells [10]. Patients who received IFN-β and demonstrated high LINE-1 methylation levels were found to be less likely to respond adequately to immunomodulatory therapy [11]. Hypermethylated LINE-1 fragments were identified in the cell-free circulating DNA of MS patients [12]. A review of DNA methylation studies in MS indicates that LINE-1 methylation shows potential as a diagnostic and prognostic biomarker, correlating with neurological deficit severity and therapeutic response [13].

The one-carbon metabolism is known to be closely associated with the maintenance of appropriate methylation levels [14]. The interplay of two coordinated cycles – the folate cycle and the homocysteine- methionine cycle – results in the production of S-adenosylmethionine (SAM), a universal methyl group donor, and S-adenosylhomocysteine (SAH), an inhibitor of DNA methyltransferase. The equilibrium of these one-carbon metabolism intermediates may be compromised in cases of dietary methionine deficiency and deficiencies in B vitamins, which function as coenzymes in homocysteine remethylation reactions. Variations in the genes for methylenetetrahydrofolate reductase (MTHFR), methionine synthase (MTR), and methionine synthase reductase (MTRR) may affect the activity of these enzymes, which are critical to the folate cycle. Consequently, changes in genomewide methylation levels can occur because of the slow conversion of homocysteine to methionine. The resultant effects may include an increased accumulation of homocysteine in the blood and variations in the SAM/SAH ratio [15].

The purpose of this study was to evaluate the LINE-1 methylation status in the peripheral blood cells of individuals with multiple sclerosis and to assess laboratory indicators of folate metabolism, specifically serum homocysteine, cyanocobalamin (vitamin B12), and folic acid (vitamin B9). Moreover, we aimed to identify genotypes for significant polymorphisms within folate cycle genes and investigate the relationship between LINE-1 methylation and folate metabolism.

## EXPERIMENTAL PART

Twenty-seven patients were recruited for this study, including twenty-three diagnosed with MS according to the 2005, 2010, and 2017 McDonald criteria [16, 17], and four patients with clinically isolated syndrome (CIS) and probable MS. Eleven patients presented a disease duration of no more than one year, while sixteen patients had had MS for a period ranging from one to twenty-three years. All the patients were under outpatient observation at the clinic of the Almazov National Medical Research Center of the Ministry of Health of the Russian Federation and the clinic of Pavlov First Saint Petersburg State Medical University. The control group comprised twenty individuals with no neurological pathology. *Table 1* summarizes the characteristics of the examined groups. Neurological impairment was assessed using the Expanded Disability Status Scale (EDSS). The rate of disease progression was assessed using the Multiple Sclerosis Severity Score (MSSS), which was calculated based on age, disease duration, and level of disability [18]. Voluntary informed written consent was obtained from all patients and healthy volunteers included in the study.

**Table 1:** Characteristics of the patients and healthy participants involved in the research

Parameter	Control (n = 20)	MS (n = 27)
Age, years	31.0 [24.5; 39.3]	33.0 [27.5; 42.5]
Sex (F : M)	16 : 4	18 : 9
EDSS, score	–	3.0 [2.0; 3.9]^*^
MSSS, score	–	3.0 [2.1; 4.1]^*^
Diagnosis and MS course (CIS/RRMS/SPMS/PPMS)	–	4/18/3/2
MS Duration, years	–	6.5 [2.8; 14.0]^*^

Note: the Age, EDSS, and MSSS data are presented as median [1st quartile; 3rd quartile].

^*^The median and interquartile ranges of EDSS, MSSS, and disease duration were assessed in patients with a disease duration exceeding one year.

**Preliminary sample preparation for methylation analysis**

Peripheral blood mononuclear cells (PBMCs) were obtained by gradient centrifugation with Ficoll from venous blood samples drawn into vacuum tubes with an anticoagulant (EDTA). DNA was extracted from the PBMC suspension using a column method with a nucleic acid isolation reagent kit (Biolabmix, Russia), in accordance with the manufacturer’s protocol. To evaluate the quality of the isolated DNA, we measured its concentration and the A260/280 absorbance ratio using a NanoDrop LITE spectrophotometer (Thermo Fisher Scientific, USA). For bisulfite conversion, the BisQuick reagent kit (Eurogen, Russia) was employed, with a minimum of 100 ng of DNA used in the reaction.

**LINE-1 methylation level**

LINE-1 methylation levels were assessed using methyl- sensitive high-resolution melting curve analysis (MS-HRM assay). PCR was performed following the amplification protocol and oligonucleotide primers as specified in [19]. Amplification, detection of fluorescent signals, and subsequent analysis of melting curves were conducted using a DT-prime detection amplifier (DNA-Technology, Russia). The PCR was performed using a final volume of 25 μL, with a prepared reaction mixture that included the SYBER Green intercalating dye (Eurogen), 20 pmol of each primer, and 10 ng of a bisulfite-modified DNA matrix. All the reactions were performed twice.

**Fig. 1:**
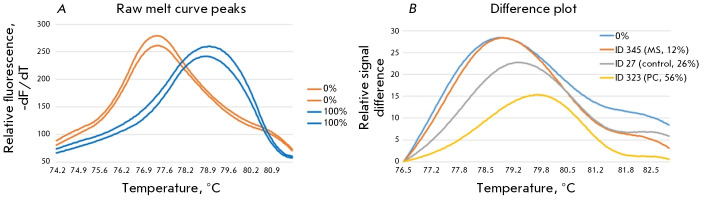
Assessment of LINE-1 methylation levels via methyl-sensitive high-resolution melting curve analysis (MS-HRM). (A) – raw melting curves and melting peaks of fully methylated (100%) and fully unmethylated (0%) standard samples; (B) – melting curves for the standard sample (0%) and the three tested samples with high (56%), low (12%), and medium (26%) methylation levels, converted to difference plots

Calibration curves were created using samples that were prepared to contain methylation percentages between 0% and 100%. Fully methylated DNA was prepared using CpG-methylase M.SSI (SibEnzyme, Russia) from the genomic DNA of human cell line L68 (SibEnzyme). Fully unmethylated DNA was represented by a human unmethylated DNA standard (CpGenome Human Non-Methylated DNA Standard Set, Sigma-Aldrich, Sweden). Samples of 100% methylated and unmethylated control DNA underwent bisulfite conversion (along with the tested samples). Next, the target fragment was amplified using the converted standard samples as a matrix. The standards were then concentration-aligned so that the difference in Ct threshold cycle values remained under two during real-time fluorescence signal detection. Subsequently, the prepared fully methylated and unmethylated standard samples were combined in specific ratios to generate calibrators with methylation levels of 25%, 50%, and 75%. Following this, all calibration samples underwent amplification at each stage. The disparate melting profiles and temperature differentials for the melting peaks of methylated and unmethylated DNA indicated amplification of the products, which are differ in the cytosine to the thymine ratio (*Fig. 1A*).

To quantify the methylation levels, the data from fluorescence measurements obtained at each point along the temperature gradient for calibrators and test samples were imported into Excel as a text file. The data were normalized, and, subsequently, a plot of the differences was generated for each normalized melting curve and compared to the baseline melting curve (corresponding to the 100% methylated standard sample). The area under the curve (AUC), representing the derivative of the HRM melting curve, was determined for post-processing MS-HRM data. After normalization on the difference plot, each curve was displayed as it appeared when the AUC value for the baseline was subtracted. Examples of difference plots for samples with different methylation levels are shown in *Fig. 1B*. The methylation levels of the samples were computed by comparing their AUC values with the calibration curve derived from standard samples with known methylation levels, according to the recommendations outlined in [20].

**Analysis of folate metabolism parameters**

Blood samples were obtained from patients and healthy donors in the morning, under fasting conditions, adhering to established pre-analytical pro tocols. The serum levels of homocysteine, folic acid (vitamin B9), and cyanocobalamin (vitamin B12) were assessed immediately following blood collection (the samples were not retained). All studies were conducted in a clinical diagnostic laboratory setting. The folic acid content was measured using an Alisei Q.S. (Next Level S.R.L., Italy) enzyme immunoassay analyzer. Cyanocobalamin was measured via chemiluminescent immunoassay, using the Alinity i analyzer (Abbott Laboratories, USA), with homocysteine determined using the Roche Cobas 6 000 automated modular platform, employing the immunochemical module e601 (Roche Diagnostics, Switzerland).

**Genotyping for polymorphisms C677T and A1298C of the *MTHFR *gene, A2756G of the *MTR *gene, and A66G of the *MTRR *gene**

Genotyping was performed by PCR using oligonucleotide primers and fluorescently labeled allele-specific probes (DNA-Synthesis, Russia). The primer and probe sequences are detailed in [21]. Genomic DNA was isolated from whole blood by the standard method using the “DNA-Sorb B” kit (AmpliSens, Russia). Statistical processing of the data was performed using the Statistica (v. 10) program package**. **The statistical criteria were selected depending on whether the data met the standards of the normal distribution.

## RESULTS

**Methylation of LINE-1 in patients with MS and in control subjects**

**Fig. 2:**
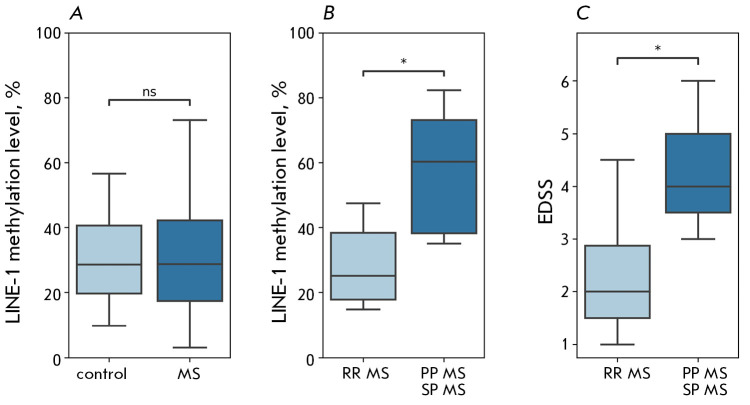
LINE-1 methylation levels in peripheral blood mononuclear cells. (A) – comparison of indices in the control group and in the group of MS patients; (B) – comparison of indices in patients with RR MS, PP MS, and SP MS; (C) – degree of neurologic deficit in patients with remitting and progressive MS. * – statistically significant differences between groups; p < 0.05; ns – no significant differences

The study examined the level of global genomic methylation in two groups: control and MS patients. Furthermore, we evaluated how the progression of the disease impacted LINE-1 methylation levels by comparing individuals with remitting-relapsing MS (RR MS) to those with progressive MS (secondary progressive and primary progressive MS). The comparison of the control group and the MS patient group without accounting for disease duration and course type revealed no significant differences (Kruskal– Wallis test, H = 2.002; *p *= 0.966) (*Fig. 2A*). However, patients with progressive forms of MS exhibited a significantly higher methylation level compared to patients with the relapsing-remitting course (Mann– Whitney U test, *p *= 0.023) (*Fig. 2B*). In patients with progressive MS, the EDSS scores, which characterize the level of neurological deficit according to the expanded Kurtzke Disability Scale, were notably higher than in patients with remitting MS types. The medians and interquartile ranges for the groups were 4.0 [3.5; 5.0] and 2.0 [1.5; 3.0] points, respectively (Mann– Whitney U test, *p* = 0.012) (*Fig. 2B*).

**Effect of age and disease duration on LINE-1 methylation levels**

**Fig. 3:**
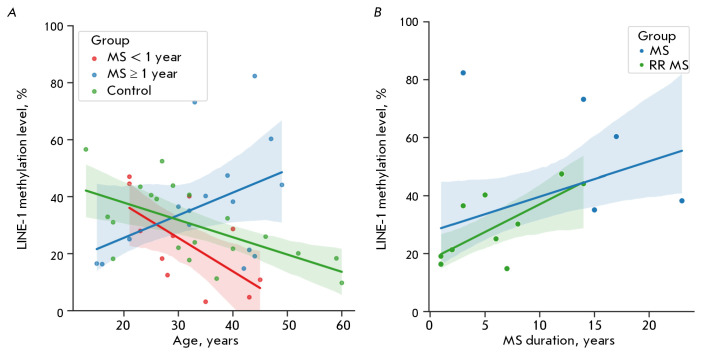
Correlation analysis data to detect changes in LINE-1 methylation levels. (A) – data from both MS patients and those in the control group; (B) – the blue line shows the correlation between the LINE-1 methylation level and disease duration for the whole group of patients, and the green line shows the results of the analysis of patients with a remitting course (RR MS) and duration of over 1 year

The study groups were similar in age, with a median age and interquartile range of 31.0 [24.5; 39.3] years in the control group and 33.0 [27.5; 42.5] years in the MS group. A significant negative correlation was found between the LINE-1 methylation level and age in the control group (*r *= -0.61; *p *= 0.004) (*Fig. 3A*). In MS patients, the significant relationship between these parameters was maintained at a similar level (*r *= -0.65;* p *= 0.032) only in those with a disease duration of less than 1 year. In patients with a longer disease duration, this correlation was absent, indicating a disruption of methylation control mechanisms in MS. Conversely, an increase in disease duration was associated with an elevation in the methylation level, as confirmed by correlation analysis revealing a positive relationship between MS duration and methylation level (*r *= 0.47;* p *= 0.014) (*Fig. 3B*). When the analysis was conducted separately in the group of patients with relapsing-remitting MS (RRMS) and excluded patients with a disease duration of less than 1 year, this trend persisted and manifested itself as a strong positive correlation (*r* = 0.72; *p* = 0.013) (*Fig. 3B*). Due to the limited number of observations (*n *= 5), a separate analysis could not be performed for patients with progressive MS. Thus, the lowest values of methylation level were observed in patients with MS duration of less than 1 year (patients at the stage of disease onset); as the disease progressed, the methylation level increased. In patients with primary progressive and secondary progressive courses of MS the methylation level was the highest. Interestingly, in patients with RRMS, the increase in methylation with longer disease duration was not associated with a rise in disability as assessed by the EDSS scale (no correlation was found between MS duration and EDSS score: *r *= -0.27; *p *= 0.452).

**LINE-1 methylation levels and folate metabolism**

To determine the mechanisms contributing to methylation dysregulation in MS, the levels of homocysteine and B vitamins were measured and the ratios of homocysteine to folic acid (Hcy/B9) and homocysteine to cyanocobalamin (Hcy/B12) were calculated in the groups under investigation. The values for every indicator analyzed are given in *Table 2*. Additionally, the polymorphisms of folate cycle genes – C677T and A1298C of the *MTHFR *gene, A2756G of the *MTR* gene, and A66G of the *MTRR *gene – were genotyped for all the subjects.

**Fig. 4:**
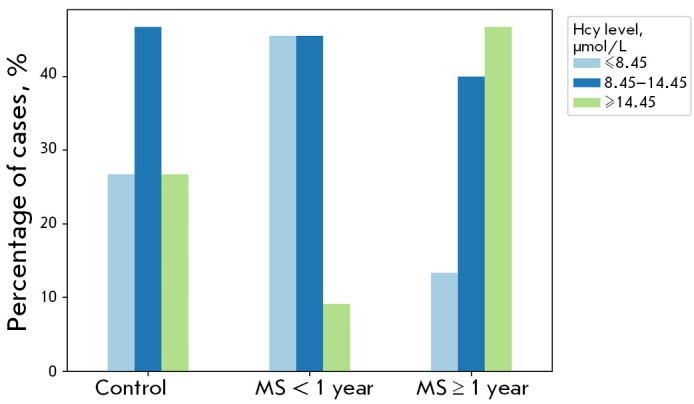
Homocysteine serum levels in the control subjects and MS patients. The concentration ranges correspond to the quartiles established for the control group. The data for each range are expressed as percentages of the entire cohort within their respective groups

Our earlier work identified the changes in folate metabolism parameters specific to the initial stage of MS (notably, decreased homocysteine levels at adult MS onset) [21]. Hence, in this work, we evaluated folate metabolism parameters across all MS patients, with a separate analysis for those with disease durations of less than and greater than a year. Using this approach, we found that in the group of patients with MS, those with a duration of less than 1 year had predominantly low and medium values of homocysteine levels. In contrast, high and medium values were predominant in the prolonged course (*Fig. 4*). Homocysteine levels relative to the lower quartile (≤ 8.45 μmol/L) and upper quartile (≥14.45 μmol/L) in the control group were taken as low and high levels.

**Table 2:** Levels of homocysteine (Hcy), folic acid (vitamin B9), vitamin B12, and the Hcy/B9 ratio in individuals with multiple sclerosis (MS) compared to a control group

Parameter, units	Control (n = 20)	All MS patients (n = 27)	MS Duration		Reference interval
< year (n = 11)	≥ year (n = 16)
Homocysteine, µmol/L	11.1 [8.5; 14.5]	11.7 [8.0; 14.8]	9.9 [6.35; 11.7]	13.5 [10.0; 15.5]	Men: 5.46–16.20 Women: 4.44–13.56
Vitamin B9, nmol/L	14.95 [12.0; 18.5]	12.6 [8.0; 27.0]	26.1 [15.1; 31.7]	8.36^#*^ [6.1; 11.1]	7.0–46.4
Vitamin B12, pg/mL	277 [211; 392]	363 [264; 551]	300 [233; 500]	375 [277; 553]	197.0–771.0
Hcy/B9	0.693 [0.455; 1.244]	0.981 [0.251; 1.7]	0.271 [0.207; 0.719]	1.724^#^ [1.129; 2.144]	

Note: the data are presented as median [1st quartile; 3rd quartile].

^#^Significant differences between the MS patient subgroups with different disease durations (Kruskal–Wallis test with subsequent pairwise comparison).

^*^Significant difference from the control group (Mann–Whitney U test).

**Fig. 5:**
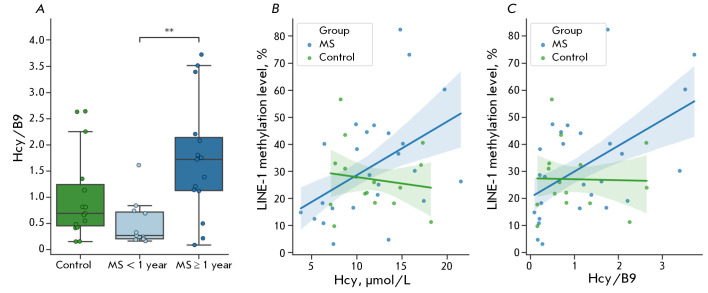
Alterations in the ratio of serum homocysteine and folic acid concentrations (Hcy/B9) in individuals with MS (A), and the correlation between alterations in homocysteine levels (B) and Hcy/B9 ratios (C) with LINE-1 methylation levels in peripheral blood mononuclear cells. ** – statistically significant differences between the groups, p < 0.05 (Kruskal– Wallis test with subsequent pairwise comparisons)

Significant differences in the Hcy/B9 ratio were also identified in patients with different durations of multiple sclerosis (MS). In patients during the initial disease period, the median Hcy/B9 ratio was 0.271, whereas in those with a prolonged disease course, it was 1.724 (*p* = 0.007). This parameter stood at an intermediate value of 0.693 in the control group (*Fig. 5A*). The primary factor in the decline of this index during the early stages of the condition (in the group of patients with multiple sclerosis duration of less than 1 year) was the reduction in homocysteine levels. In contrast, folic acid levels remained normal, with all patients in this group exhibiting values within the established reference range (7.0–46.4 nmol/L). Throughout the protracted course of MS, a significant decline in B9 content was observed relative to the control group (Mann–Whitney U test, *p *= 0.024) and the vitamin B9 concentration was at or below the lower threshold of the reference interval in 10 out of 16 patients. Conversely, the level of homocysteine in patients with chronic MS tended to be high, with three patients presenting hyperhomocysteinemia (homocysteine concentrations exceeding 13.56 μmol/L in women and 16.20 μmol/L in men). Therefore, elevated Hcy/B9 values during the prolonged course of MS stemmed from heightened homocysteine levels and reduced vitamin B9 levels.

Furthermore, a significant positive correlation was found in MS patients but not in the control group between the level of methylation and homocysteine content (*r *= 0.45; *p *= 0.020), as well as between the level of methylation and the Hcy/B9 ratio (*r *= 0.52;* p *= 0.006) (*Fig. 5B,C*). Regression analysis indicated that the Hcy/B9 ratio could be a predictor of the methylation level (*p* = 0.010).

**Fig. 6:**
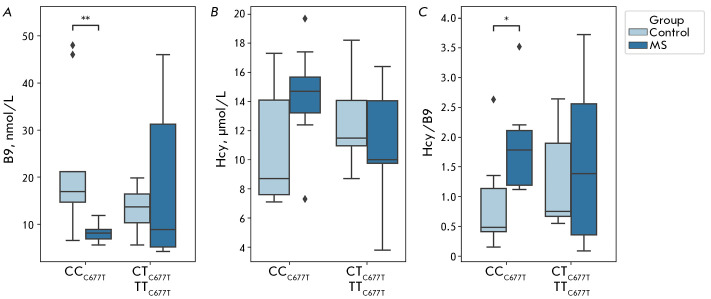
Concentration of vitamin B9 (A) and homocysteine (B), and the Hcy/B9 ratio (C) in the individuals of the control group and the MS patients depending on the genotype for the C677T polymorphism of the MTHFR gene. ♦ – values deviating from the median by more than 1.5 interquartile range. * – statistically significant differences between the groups, p < 0.05; ** – statistically significant differences between the groups, p < 0.01

Regression analysis did not reveal a statistically significant contribution of the studied polymorphic variants of folate cycle genes to changes in LINE-1 methylation levels. Conversely, a notable decrease in vitamin B9 levels, when compared to the control group, was only observed in patients with the CC genotype based on the C677T polymorphism of the* MTHFR *gene, but not in carriers of the minor T allele (*Fig. 6A*). This analysis was limited to the patient cohort with MS duration exceeding 1 year. Individuals with the CC genotype also tended to have higher homocysteine concentrations (*Fig. 6B*) and a notable increase in the Hcy/B9 ratio (*Fig. 6C*).

The patterns observed suggest the influence of the C677T polymorphism of the *MTHFR *gene on folate metabolism-mediated impairment of methylation control in patients with MS.

## DISCUSSION

Evaluation of the methylation level of the LINE-1 retrotransposon serves as a surrogate marker of global genomic DNA methylation, as these repetitive genetic elements represent up to 70% of the methylated CpG sites within the genome [22, 23]. Alterations in LINE-1 methylation are also noteworthy, given that epigenetic silencing of retrotransposons may contribute to genome instability, chromosomal structural rearrangements, and malignization [24, 25]. In elderly individuals, the activation of LINE-1 retrotransposons results in the induction of interferon synthesis and contributes to the stimulation of inflammatory responses [26].

The present study revealed significant differences in the methylation level of the LINE-1 retrotransposon in PMNCs in patients with remitting and progressive MS, with higher methylation values found in patients with progressive MS.

Furthermore, LINE-1 methylation levels were observed to rise in multiple sclerosis patients, correlating with the length of their disease. In the control group, however, the level of methylation was inversely correlated with age. More precisely, MS alters correlation patterns, such as the loss of the negative correlation between age and global genomic methylation observed in healthy individuals, which is absent in patients.

Altered levels of homocysteine, folic acid, and their ratio in MS patients, coupled with the correlation between these changes and LINE-1 methylation levels, point to potential folate metabolism disruptions as a cause of hypermethylation in MS. One-carbon metabolism is a multi-component metabolic process that occurs in multiple steps. S-adenosylmethionine (SAM) and S-adenosylhomocysteine (SAH), which are intermediaries in the homocysteine-methionine cycle, modulate the activity of DNA methyltransferase, with stimulatory (SAM) and inhibitory (SAH) effects [27, 28]. The imbalance between these metabolites may be attributed to several factors, such as dietary methionine irregularities, deficiency cofactors of folate-metabolizing enzymes, and the presence of gene polymorphisms associated with homocysteine remethylation. Under normal physiological conditions, homocysteine is readily processed, rendering its creation biochemically advantageous. However, the accumulation of homocysteine shifts the equilibrium towards the preferential formation of SAH, a potent inhibitor of methyltransferase activity due to its structural similarity to the SAM molecule [29, 30]. The DNA methyltransferase DNMT1, which is essential for maintaining methylation patterns during cell division, is particularly sensitive to the inhibitory action of SAH. Consequently, an augmentation in blood homocysteine concentration should be correlated with hypomethylation, a phenomenon substantiated in several studies [31, 32, 33, 34]. However, in patients with MS, the elevated homocysteine level was associated with increased methylation, suggesting a disruption in the feedback mechanism governing DNMT activity. This suggestion necessitates supplementary research for confirmation.

It is worth noting that only four out of 27 patients exhibited homocysteine levels exceeding the upper limit of the reference range, with a peak value of 21.5 μmol/L in one patient, indicating a moderate degree of hyperhomocysteinemia. The elevation in homocysteine concentration may be insufficient to shift the equilibrium toward SAH formation, thereby failing to inhibit methyltransferase activity.

The observed changes in patients with multiple sclerosis may be attributed to the dysregulation of the MTHFR enzyme, which is responsible for the conversion of 5,10-methylenetetrahydrofolate to 5-methyltetrahydrofolate, the adequate production of which is crucial for the synthesis of SAM and the availability of methyl groups. MTHFR is allosterically inhibited by SAM [35]. The absence of this mechanism results in 5-methyltetrahydrofolate being consistently produced, thereby facilitating increased homocysteine remethylation, methionine biosynthesis independent of its concentration, and SAM synthesis [36]. Our findings may be indicative of the realization of such a mechanism. Thus, throughout the chronic progression of MS, an elevation in the percentage of patients exhibiting high serum homocysteine levels (relative to the average in the control group) was observed, with more frequent indications of folate deficiency and a predictable increase in the Hcy/B9 ratio. An examination of these parameters in relation to the C677T polymorphism of the *MTHFR *gene genotype revealed a greater propensity for these changes in individuals with the CC genotype. Alterations in folate metabolism demonstrated a correlation with methylation level changes. Therefore, it is reasonable to hypothesize that the influence of the C allele of this polymorphic variant contributes to the manifestation of epigenetic disorders in the development of MS. The hypothesis is supported by the finding that a missense mutation at position 677 of the *MTHFR *gene causes a decrease in enzyme activity [37]. MTHFR catalyzes the conversion of tetrahydrofolate to 5-methyltetrahydrofolate, providing a substrate for the MTR-mediated remethylation of homocysteine to methionine. The presence of the T allele of the C677T polymorphism in the *MTHFR *gene lowers enzyme activity by a maximum of 70% in heterozygous and 30% in homozygous carriers. Consequently, a marked reduction in vitamin B9 levels among carriers of the “active” gene variant (genotype CC) might be linked to its increased utilization in the process of converting homocysteine to methionine, maintaining methionine concentrations, facilitating SAM formation, and sustaining a high methylation potential. The idea presented here is congruent with prior findings, which suggest that individuals with the TT genotype of the C677T polymorphism exhibit decreased global methylation in lymphocyte DNA [38]. Additionally, the effect of hyperhomocysteinemia on peripheral mononuclear cell methylation is determined by the C677T polymorphism genotype of the *MTHFR *gene and folate levels: a reduction in methylcytosine levels was noted in individuals with the TT genotype and diminished blood folate [39].

## CONCLUSIONS

The present study is the first to demonstrate a connection between LINE-1 methylation levels in multiple sclerosis and the folate metabolism status. Overall, the obtained results are in good agreement with the current understanding of the influence of metabolic processes on the key epigenetic phenomenon – DNA methylation. One-carbon fragment metabolism disorders can be triggered by inadequate vitamin and nutrient intake or by polymorphic variations in the genes involved in the folic acid and homocysteine remethylation processes. The development of aberrant methylation patterns and persistent alterations in gene expression is attributable to diminished methyl donor availability and dysregulated methyltransferase activity, both consequences of an impaired folate metabolism. It should be emphasized that epigenetic alterations are regulated and can be reversed. Future research endeavors should prioritize the development of algorithms to correct metabolic disorders and maintain sufficient methylation levels.

## Abbreviations

MS: multiple sclerosis
CIS: clinically isolated syndrome
CNS: central nervous system
LINE1: Long Interspersed Nuclear Element-1
BBB: blood-brain barrier
SAM: S-adenosylmethionine
SAH: S-adenosylhomocysteine
MTHFR: methylenetetrahydrofolate reductase
MTR: methionine synthase
MTRR: methionine synthase reductase
Hcy: homocysteine
Hcy/B9: the ratio of homocysteine levels to vitamin B9 levels
PMNC: peripheral blood mononuclear cells
EDTA: ethylenediaminetetraacetic acid
EDSS: Expanded Disability Status Scale
MSSS: Multiple Sclerosis Severity Score
MS-HRM analysis: Methyl-sensitive High-Resolution Melting Assay
PCR: polymerase chain reaction
AUC: Area Under Curve
RR MS: relapsing-remitting multiple sclerosis
PP MS: primary progressive multiple sclerosis
SP MS: secondary progressive multiple sclerosis
